# Low Serum C-Peptide and Albuminuria Severity in Type 2 Diabetes Mellitus: A Cross-Sectional Study

**DOI:** 10.3390/jcm15186950

**Published:** 2026-09-08

**Authors:** Bektas Isik, Bekir Tamer Tetiker

**Affiliations:** 1 Department of Internal Medicine, Adana Health Practice and Research Center, University of Health Sciences, 01230 Adana, Türkiye; 2 Division of Endocrinology and Metabolism, Department of Internal Medicine, Çukurova University Faculty of Medicine, 01790 Adana, Türkiye

**Keywords:** albuminuria, beta-cell function, c-peptide, clinical research, cross-sectional study, diabetic complications, diabetic kidney disease, type 2 diabetes mellitus

## Abstract

**Background/Objectives:** Lower serum C-peptide, reflecting reduced beta-cell reserve, has been linked to diabetic complications, but whether these associations are independent of diabetes duration and glycaemic control is unclear. The objective of this study was to determine which microvascular and macrovascular complications of type 2 diabetes mellitus (T2DM) remain associated with fasting C-peptide after adjustment for these factors. **Methods:** In this single-centre, cross-sectional study with consecutive prospective enrolment, non-pregnant adults with an established diagnosis of T2DM attending routine outpatient follow-up were eligible; those with pancreatic malignancy or exocrine pancreatic insufficiency, monogenic or autoimmune diabetes, or acute hyperglycaemic crises were excluded. A total of 590 patients were stratified by fasting C-peptide into insufficient (<1.0 ng/mL; *n* = 35), borderline (1.0–1.8 ng/mL; *n* = 212) and normal/high (≥1.8 ng/mL; *n* = 343) groups. Logistic regression adjusted for age, sex, diabetes duration, HbA1c and body mass index assessed each complication. Receiver operating characteristic analysis using DeLong’s test assessed incremental discrimination. **Results:** In the unadjusted analyses, every complication was more frequent at lower C-peptide. After adjustment, C-peptide was not associated with the presence of albuminuria (insufficient versus normal/high, odds ratio 1.60, 95% CI 0.16–15.86) but was strongly associated with its severity: for severely increased or nephrotic-range albuminuria, the adjusted odds ratios were 13.47 (5.33–34.07) and 6.90 (3.83–12.44) in the insufficient and borderline groups (both *p* < 0.001). Adding C-peptide to a model of diabetes duration, HbA1c and fasting glucose was associated with a statistically significant, but modest, improvement in discrimination for severe albuminuria (area under the curve of 0.748 to 0.783; ΔAUC = 0.035, *p* = 0.027). Associations with macrovascular complications were present in the unadjusted analyses but were substantially attenuated after adjustment and no longer statistically significant, except for peripheral arterial disease and foot ulcer, which remained associated with insufficient C-peptide in models based on small numbers of events and should be regarded as exploratory. **Conclusions:** Low C-peptide was independently associated with the severity of albuminuria rather than its presence and was associated with a modest incremental improvement in discrimination. These exploratory findings suggest that C-peptide, an inexpensive and widely available measurement, may provide information associated with advanced diabetic kidney disease; prospective, externally validated studies are needed before clinical application.

## 1. Introduction

Type 2 diabetes mellitus (T2DM) is a chronic metabolic disorder characterised by sustained hyperglycaemia arising from a progressive decline in beta-cell insulin secretion superimposed on insulin resistance [1]. Its global burden continues to rise: the International Diabetes Federation estimated that 588.7 million adults were living with diabetes in 2024, a figure projected to reach 852.5 million by 2050 [2]. The clinical importance of T2DM derives not only from chronic hyperglycaemia but, to an even greater extent, from its complications, which are key determinants of patient morbidity and mortality.

The chronic complications of diabetes are conventionally divided into microvascular disease—retinopathy, nephropathy and neuropathy—and macrovascular disease, which includes coronary artery disease, cerebrovascular disease and peripheral arterial disease [1]. Microvascular complications result from chronic hyperglycaemia-induced damage to the small vessels and capillaries supplying the retina, renal glomeruli and peripheral nerves, whereas macrovascular disease reflects accelerated atherosclerosis of larger arteries. Both categories share a pathophysiological background of endothelial dysfunction, oxidative stress and chronic low-grade inflammation, and acute complications, such as diabetic ketoacidosis and the hyperglycaemic hyperosmolar state, add to the overall disease burden [3,4]. Notably, microvascular dysfunction is detectable even before the diagnostic threshold for diabetes is crossed, with impaired microcirculatory reactivity already evident in prediabetes [5], indicating that the vascular processes discussed here begin earlier in the disease course than the diagnosis of T2DM itself.

C-peptide, a 31-amino-acid by-product of proinsulin cleavage co-secreted with insulin in equimolar amounts, is more stable and less subject to first-pass hepatic extraction than insulin, making it a practical marker of residual beta-cell function [6,7]. Once regarded as a biologically inert by-product, C-peptide is now recognised as a bioactive peptide that influences microvascular perfusion, endothelial integrity and inflammatory signalling [6]. Clinically, basal C-peptide has been proposed as a surrogate marker of subclinical atherosclerosis [8], 2 h serum C-peptide has been associated with carotid intima–media thickness [9], and lower C-peptide concentrations have been linked to cardiovascular autonomic neuropathy, assessed by standard cardiovascular autonomic reflex testing, in T2DM [10]. The pathogenesis of hyperglycaemia-related cardiovascular and autonomic complications is thought to involve activation of protein kinase C (PKC) and mitogen-activated protein kinase (MAPK) signalling, which has been implicated in cardiac sympathetic overactivation in T2DM [11]. Collectively, these observations point to a close, though still incompletely defined, relationship between C-peptide and diabetic complications.

A recurring difficulty in the literature is that low C-peptide is closely correlated with long diabetes duration and poor glycaemic control, both of which are themselves powerful determinants of end-organ damage. Studies that report crude associations, therefore, cannot separate a specific signal from generic disease severity, and the few that adjust have reached inconsistent conclusions about which complications retain an independent link. Against this background, we conducted a cross-sectional, observational study with consecutive prospective enrolment to evaluate the relationship between fasting serum C-peptide levels and the prevalence and severity of microvascular and macrovascular complications in patients with T2DM. We hypothesised that lower C-peptide levels, reflecting reduced endogenous insulin secretion, would be associated with a greater burden of diabetes-related complications. The objective of this study was to determine, among patients with T2DM, which of these associations remain independent of diabetes duration and glycaemic control after adjustment.

## 2. Materials and Methods

### 2.1. Study Design and Setting

This single-centre, cross-sectional, observational study with consecutive prospective enrolment was conducted at the Internal Medicine Outpatient Clinic of Yayladağı State Hospital (Hatay, Türkiye) between 18 December 2025 and 18 February 2026. The protocol was approved by the Non-Interventional Clinical Research Ethics Committee of Hatay Mustafa Kemal University Faculty of Medicine (meeting no. 16, decision no. 42, dated 17 December 2025), and this study was conducted in accordance with the Declaration of Helsinki [12]. Written informed consent was obtained from all participants. As a purely observational study in which the assignment of any medical intervention was not at the discretion of the investigators, this study did not require registration in a public clinical trials registry. Reporting follows the STROBE recommendations for observational studies; a completed STROBE checklist is provided as Supplementary Table S3.

### 2.2. Participants

Consecutive non-pregnant adults (aged > 18 years) with an established diagnosis of T2DM who presented for routine outpatient follow-up during the study period were eligible, irrespective of known renal status at presentation. Patients with pancreatic malignancy or exocrine pancreatic insufficiency, pregnant patients, those with monogenic diabetes (MODY) or latent autoimmune diabetes in adults (LADA), and those presenting with diabetic ketoacidosis or hyperglycaemic hyperosmolar state were excluded, as these conditions may confound C-peptide and insulin measurements.

### 2.3. Data Collection

For each participant, we recorded demographic data—age, sex and body mass index (BMI)—and duration of diabetes, retrieved from the hospital’s electronic health record system (Fonet Hastane Bilgi Yönetim Sistemi [HBYS]; Fonet Bilgi Teknolojileri, Ankara, Türkiye). Fasting blood samples were analysed for serum C-peptide, glucose, insulin, glycated haemoglobin (HbA1c), blood urea nitrogen, creatinine, total cholesterol, low-density lipoprotein (LDL) cholesterol, high-density lipoprotein (HDL) cholesterol, triglycerides and magnesium. The estimated glomerular filtration rate (eGFR) was calculated using the CKD-EPI equation. Antidiabetic and concomitant treatments were documented, including single or multiple oral antidiabetic drugs (OADs), OADs plus basal insulin, OADs plus basal–bolus insulin, basal–bolus insulin, basal insulin alone, angiotensin-converting enzyme inhibitors or angiotensin receptor blockers (ACEi/ARBs), statins and sodium–glucose co-transporter-2 (SGLT-2) inhibitors.

### 2.4. Biochemical Measurements

Blood samples for C-peptide, insulin, glucose and the other biochemical parameters listed above were drawn after a minimum 8 h overnight fast, consistent with recommended fasting sampling protocols for the assessment of beta-cell function in type 2 diabetes [13]. In the participants treated with insulin, the sample was additionally timed so that at least 8 h had elapsed since the most recent insulin injection, to minimise acute exogenous suppression of endogenous insulin secretion and its potential effect on the measured C-peptide concentration. Serum C-peptide was measured by electrochemiluminescence immunoassay (ECLIA) on a Roche cobas 8000 modular analyser (Roche Diagnostics, Mannheim, Germany). The laboratory reference interval for fasting serum C-peptide on this platform was 0.78–1.89 ng/mL; note that the group thresholds used in this study were chosen to reflect beta-cell secretory reserve (see Section 2.6) and do not correspond to this reference interval. HbA1c was measured by an NGSP-certified high-performance liquid chromatography method, and the remaining biochemical parameters were analysed on the same platform.

### 2.5. Definition of Complications

Urinary albumin was quantified as the albumin-to-creatinine ratio (ACR) in a spot urine sample, and albuminuria was classified as normoalbuminuria (ACR < 30 mg/g), microalbuminuria (30–299 mg/g), severely increased albuminuria (300–3499 mg/g) or nephrotic-range albuminuria (≥3500 mg/g) [14]; we use albumin-based terminology throughout because only urinary albumin, not total urinary protein, was measured.

With the exception of albuminuria, all complications were ascertained from each participant’s existing medical records rather than by protocol-driven examination at the study visit; the criteria set out below are those applied in routine care at our institution when the corresponding diagnosis is established.

Diabetic retinopathy and diabetic peripheral neuropathy were not assessed de novo for this study. Both were ascertained from each participant’s existing medical records and were recorded as present when a documented diagnosis was available. In routine care these diagnoses are established according to standard criteria: for retinopathy, dilated fundus examination by an ophthalmologist [15], using grading from the International Clinical Diabetic Retinopathy Disease Severity Scale [16]; and for neuropathy, a symptom history together with 10-g Semmes–Weinstein monofilament testing, combined with at least one additional modality (vibration perception with a 128 Hz tuning fork, pinprick, temperature sensation or ankle reflexes) [15]. The individual examinations underlying each recorded diagnosis were not re-verified by the investigators.

Coronary artery disease was defined as a documented history of myocardial infarction, according to the Fourth Universal Definition of Myocardial Infarction [17], angiographically confirmed stenosis of at least 50% in one or more epicardial coronary arteries, or prior coronary revascularisation by percutaneous coronary intervention or coronary artery bypass grafting.

Cerebrovascular disease was defined as a documented, imaging-confirmed ischaemic or haemorrhagic stroke, or a physician-diagnosed transient ischaemic attack, using the American Heart Association/American Stroke Association definitions [18].

Peripheral arterial disease was ascertained from each participant’s medical history and was recorded as present when a previously established diagnosis was documented. Ankle–brachial index measurement was not performed as part of this study; in routine care, the diagnosis rests on the criteria set out in the 2024 multisociety guideline for lower-extremity peripheral artery disease, principally including a resting ankle–brachial index of 0.90 or less, imaging-confirmed lower-extremity arterial stenosis, or prior peripheral revascularisation [19].

Diabetic foot ulcer was defined, following the International Working Group on the Diabetic Foot, as a break in the skin of the foot below the level of the malleoli that penetrates the epidermis and at least part of the dermis [20], and was recorded as present for an active ulcer or a documented previous ulcer.

Hypertension was defined as a systolic blood pressure of at least 130 mmHg or a diastolic blood pressure of at least 80 mmHg, based on the average of two or more measurements obtained on two or more occasions, or current use of antihypertensive medication [21]. Blood pressure was measured with a validated upper-arm automated oscillometric device (Omron M3; Omron Healthcare Co., Ltd., Kyoto, Japan), listed among validated devices in the STRIDE BP database [22], after at least 5 min of seated rest with the arm supported at heart level.

### 2.6. C-Peptide Grouping

The participants were stratified into three groups reflecting insufficient, borderline, and normal/high beta-cell reserve, adapting the tri-level fasting C-peptide classification previously used to categorise beta-cell reserve in patients with type 2 diabetes [23]: insufficient (<1.0 ng/mL; *n* = 35), borderline (1.0–1.8 ng/mL; *n* = 212) and normal/high (≥1.8 ng/mL; *n* = 343). The upper threshold of 1.8 ng/mL (600 pmol/L) corresponds to the fasting C-peptide value above which the American Diabetes Association considers endogenous insulin secretion to be clinically preserved irrespective of sampling conditions, whereas values below this threshold may indicate reduced beta-cell reserve [1].

### 2.7. Statistical Analysis

Analyses were performed using IBM SPSS Statistics for Windows, version 27.0 (IBM Corp., Armonk, NY, USA). The moderation analysis was carried out with the PROCESS macro (Model 1) for SPSS. Firth’s penalised-likelihood logistic regression and DeLong’s test for correlated ROC curves are not implemented in SPSS; these were performed with R version 4.5.1 (R Foundation for Statistical Computing, Vienna, Austria) using the logistf package (version 1.26.1) and pROC package (version 1.19.0.1). The distribution of continuous variables was assessed by visual inspection of histograms and Q–Q plots together with skewness and kurtosis values; the Shapiro–Wilk test was not used as the primary criterion because, in a sample of this size (*n* = 590), it is highly sensitive to trivial, clinically unimportant deviations from normality and tends to reject normality even for approximately normal distributions [24]. As continuous variables were non-normally distributed, they are summarised as median (25th–75th percentile) and categorical variables as number (percentage). Continuous variables were compared across the three C-peptide groups using the Kruskal–Wallis test with the Dunn–Bonferroni post-hoc test; categorical variables were compared using the Pearson chi-square test, with Fisher’s exact test and Bonferroni correction for pairwise comparisons. For categorical comparisons, effect sizes were quantified using Cramér’s V, computed from the Pearson chi-square statistic of the corresponding C-peptide-group contingency table (V = √[χ^2^/(*n* × (k − 1))], where k is the smaller dimension of the table); values of approximately 0.1, 0.3 and 0.5 were interpreted as small, moderate and large effects, respectively. Associations between C-peptide and demographic or clinical variables were examined using Spearman’s rank correlation coefficient (ρ) and are reported in Supplementary Table S1.

Five records required checking against the source documents before analysis: two carried both a normoalbuminuria and a severely increased-albuminuria flag, two contained out-of-range codes in the statin- and antidiabetic-regimen fields, and one had a missing BMI. All five were resolved from the source records and corrected, so the analysed data set was complete for all 590 participants; no imputation was performed. No a priori sample-size calculation was performed: this study enrolled all consecutive eligible patients presenting during a fixed two-month recruitment window, so the achieved sample size (*n* = 590) was determined by this recruitment period rather than by a pre-specified power calculation.

To examine the association between medication use and albuminuria severity, ordinal logistic regression was used, treating albuminuria as a four-level ordinal outcome (normoalbuminuria < microalbuminuria < severely increased albuminuria < nephrotic-range albuminuria); the results are reported in Supplementary Table S2. Unadjusted models included ACEi/ARB and SGLT-2 inhibitor use; adjusted models additionally controlled for age, duration of diabetes, HbA1c and CKD-EPI eGFR. The proportional-odds assumption was evaluated by fitting separate binary logistic models at each cut point and comparing coefficients.

A moderation analysis (PROCESS Model 1) examined whether insulin moderated the association between C-peptide (independent variable) and HbA1c (dependent variable), with age, sex and duration of diabetes as covariates; conditional effects of C-peptide were estimated at the 16th, 50th and 84th percentiles of fasting insulin. All tests were two-tailed, and a *p*-value < 0.05 was considered statistically significant.

To determine whether C-peptide was independently associated with complications, each complication was modelled separately by multivariable binary logistic regression with C-peptide group (insufficient and borderline versus normal/high as the reference) as the exposure. Models were adjusted a priori for age, sex, duration of diabetes, HbA1c and BMI, using a clinically specified rather than data-driven (e.g., stepwise) covariate set to avoid overfitting in this pre-specified primary analysis. Insulin and eGFR were deliberately excluded from these primary models to avoid collinearity and over-adjustment for variables that may lie on or close to the hypothesised causal pathway linking beta-cell reserve to renal outcomes (all variance inflation factors ≤ 1.81). Because eGFR differed significantly across C-peptide groups and renal clearance could, in principle, influence circulating C-peptide, a sensitivity analysis additionally adjusting the principal renal severity outcome for CKD-EPI eGFR was performed to test the robustness of this exclusion (Section 3.5). Two renal outcomes were modelled: the presence of albuminuria of any category versus normoalbuminuria, and severely increased or nephrotic-range albuminuria versus normo- or microalbuminuria. Composite microvascular and macrovascular endpoints, and a Poisson model of the total number of complications, were also fitted. For complications with fewer than 30 events, and for outcomes showing quasi-complete separation, Firth’s penalised-likelihood logistic regression with limited adjustment (age and duration of diabetes) was used to reduce small-sample bias. C-peptide was additionally modelled continuously; because its distribution was strongly right-skewed, it was natural-log transformed and expressed per 1-standard-deviation decrease. Crude and adjusted odds ratios (incidence rate ratios for the count model) with 95% confidence intervals were reported.

To assess whether C-peptide provided incremental discriminatory information beyond established risk factors, receiver operating characteristic (ROC) curve analysis was performed for both renal outcomes. The area under the curve (AUC) was calculated for a base logistic regression model containing diabetes duration, HbA1c and fasting plasma glucose (standardised to z-scores), and compared with a model additionally including log-transformed C-peptide, using DeLong’s method for paired, correlated ROC curves [25]. The discriminative ability of C-peptide alone was also assessed, and the optimal C-peptide cutoff was determined using the Youden index.

## 3. Results

### 3.1. Group Characteristics

A total of 590 patients were analysed (median age 62.0 years, IQR 55.0–70.0; 368 [62.4%] female; 344 [58.3%] with hypertension). The characteristics of the three C-peptide groups are summarised in Table 1. The patients in the insufficient C-peptide group were older (*p* = 0.001) and had a longer duration of diabetes (*p* < 0.001) than those in the borderline and normal/high groups. Glycaemic control worsened progressively as C-peptide declined, with higher HbA1c and fasting plasma glucose in the insufficient group (both *p* < 0.001). Renal function was also poorer in this group: urea was higher (*p* < 0.001), creatinine was higher (*p* = 0.035) and CKD-EPI eGFR was lower (*p* = 0.001). Total cholesterol (*p* = 0.048), LDL cholesterol (*p* = 0.011) and triglycerides (*p* = 0.025) were lower in the insufficient group, whereas insulin levels increased markedly across the groups in parallel with C-peptide (*p* < 0.001). BMI, sex, HDL cholesterol, magnesium and hypertension did not differ significantly between groups.

### 3.2. Microvascular and Macrovascular Complications

In unadjusted analyses, both microvascular and macrovascular complications were more frequent among patients with lower C-peptide levels (Table 1); the independence of these associations from diabetes duration and glycaemic control is examined in Section 3.5. Across the whole cohort, 61 patients (10.3%) were normoalbuminuric, 397 (67.3%) had microalbuminuria, 102 (17.3%) had severely increased albuminuria and 30 (5.1%) had nephrotic-range albuminuria. The distribution shifted markedly toward more severe categories as C-peptide declined: severely increased and nephrotic-range albuminuria together accounted for 60.0% in the insufficient group, 40.6% in the borderline group and 7.3% in the normal/high group (*p* < 0.001; Cramér’s V 0.35). Retinopathy and neuropathy were more common with lower C-peptide (both *p* < 0.001; V 0.20–0.36). Macrovascular complications were likewise more frequent in the insufficient group, including coronary artery disease (*p* = 0.005), cerebrovascular disease (*p* = 0.002), peripheral arterial disease (*p* = 0.001), and foot ulcer (*p* < 0.001), with small-to-moderate effect sizes (V 0.13–0.21). The distribution of antidiabetic treatment differed across groups, and SGLT-2 inhibitor use varied significantly (*p* < 0.001), whereas statin and ACEi/ARB use did not.

### 3.3. Correlation and Ordinal Regression Analyses

C-peptide was inversely correlated with age, duration of diabetes, HbA1c, fasting plasma glucose, urea, albuminuria category, and the total number of complications, and positively correlated with eGFR and insulin (Spearman’s ρ −0.64 to 0.72, all *p* < 0.05; Supplementary Table S1). The strongest correlations were with insulin (ρ = 0.72) and diabetes duration (ρ = −0.64), and albuminuria category correlated inversely with C-peptide (ρ = −0.40).

In unadjusted ordinal logistic regression, both ACEi/ARB use (OR 1.74, 95% CI 1.23–2.46; *p* = 0.002) and SGLT-2 inhibitor use (OR 1.70, 95% CI 1.20–2.41; *p* = 0.003) were associated with higher odds of more severe albuminuria, a pattern consistent with confounding by indication rather than with a harmful drug effect, since both agents are preferentially prescribed to patients with established kidney disease. After adjustment for age, diabetes duration, HbA1c, and eGFR, neither association persisted (ACEi/ARB OR 1.07, *p* = 0.719; SGLT-2 inhibitor OR 1.38, *p* = 0.099), whereas diabetes duration, HbA1c, and eGFR remained strongly associated with albuminuria severity (all *p* < 0.001; Supplementary Table S2). Inspection of cut-point-specific coefficients suggested that the proportional-odds assumption held reasonably for the treatment variables but was less well satisfied for eGFR, whose association was steepest at the transition from normo- to microalbuminuria; the corresponding estimate should, therefore, be read as an average across cut points.

### 3.4. Moderation Analysis

**Figure 1:**
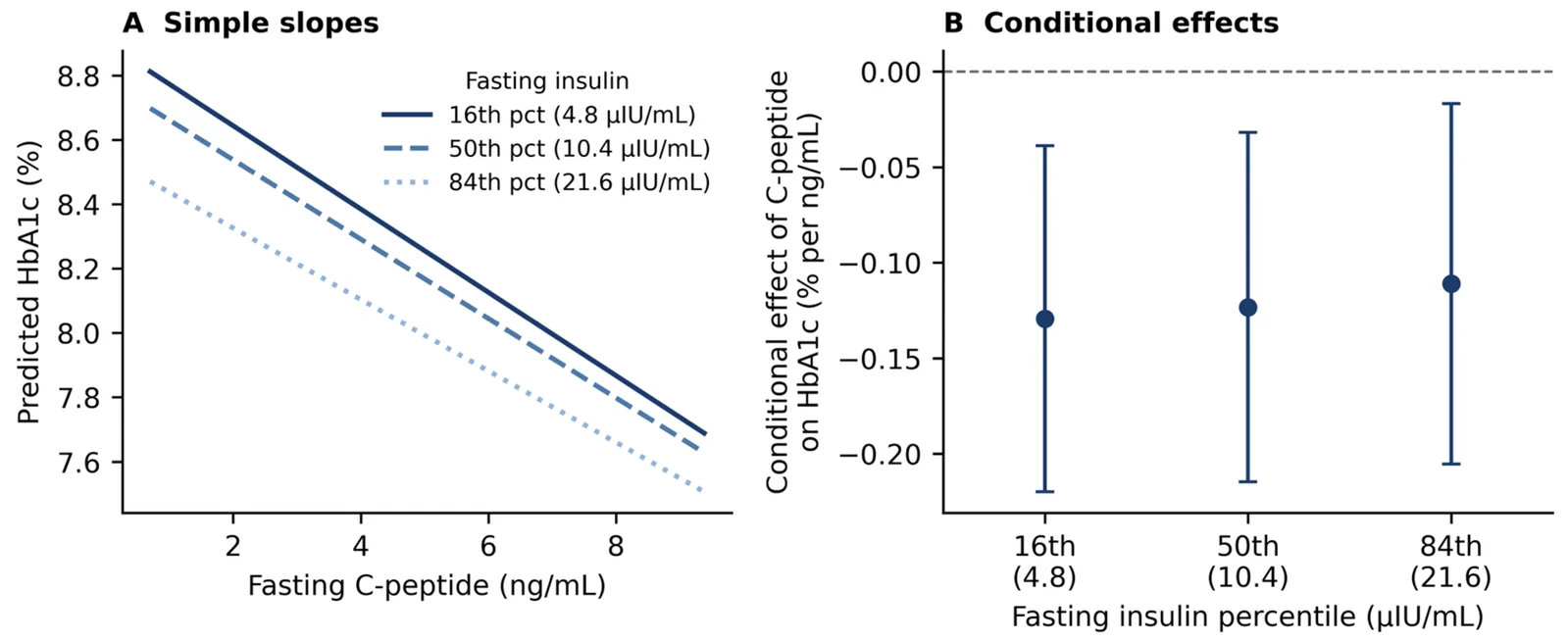
The moderating effect of fasting insulin on the association between C-peptide and HbA1c. (**A**) Model-predicted HbA1c as a function of fasting C-peptide at the 16th, 50th and 84th percentiles of fasting insulin, with age, sex and duration of diabetes held at their sample means. (**B**) The conditional effect of C-peptide on HbA1c (percentage points of HbA1c per 1 ng/mL of C-peptide) with 95% confidence intervals at the same three insulin percentiles; the dashed line marks a null effect. The interaction term was statistically significant (β = 0.0011, *p* = 0.030). HbA1c, glycated haemoglobin.

Moderation analysis (Figure 1) revealed a significant C-peptide × insulin interaction in predicting HbA1c after adjustment for age, sex and diabetes duration (β = 0.0011, *p* = 0.030). Higher C-peptide was associated with lower HbA1c (β = −0.135, *p* = 0.003), and insulin was independently associated with lower HbA1c (β = −0.021, *p* = 0.041). The inverse C-peptide–HbA1c association was strongest at lower insulin levels (β = −0.129 at the 16th percentile, *p* = 0.005) and attenuated as insulin increased (β = −0.111 at the 84th percentile, *p* = 0.021), although it remained statistically significant across all examined insulin percentiles. These findings suggest that the favourable association between C-peptide and glycaemic control is partly dependent on circulating insulin levels. The model explained a modest proportion of the variance in HbA1c (R^2^ = 0.063), so this interaction should be regarded as a statistically detectable but clinically small effect.

### 3.5. Multivariable Analysis

**Figure 2:**
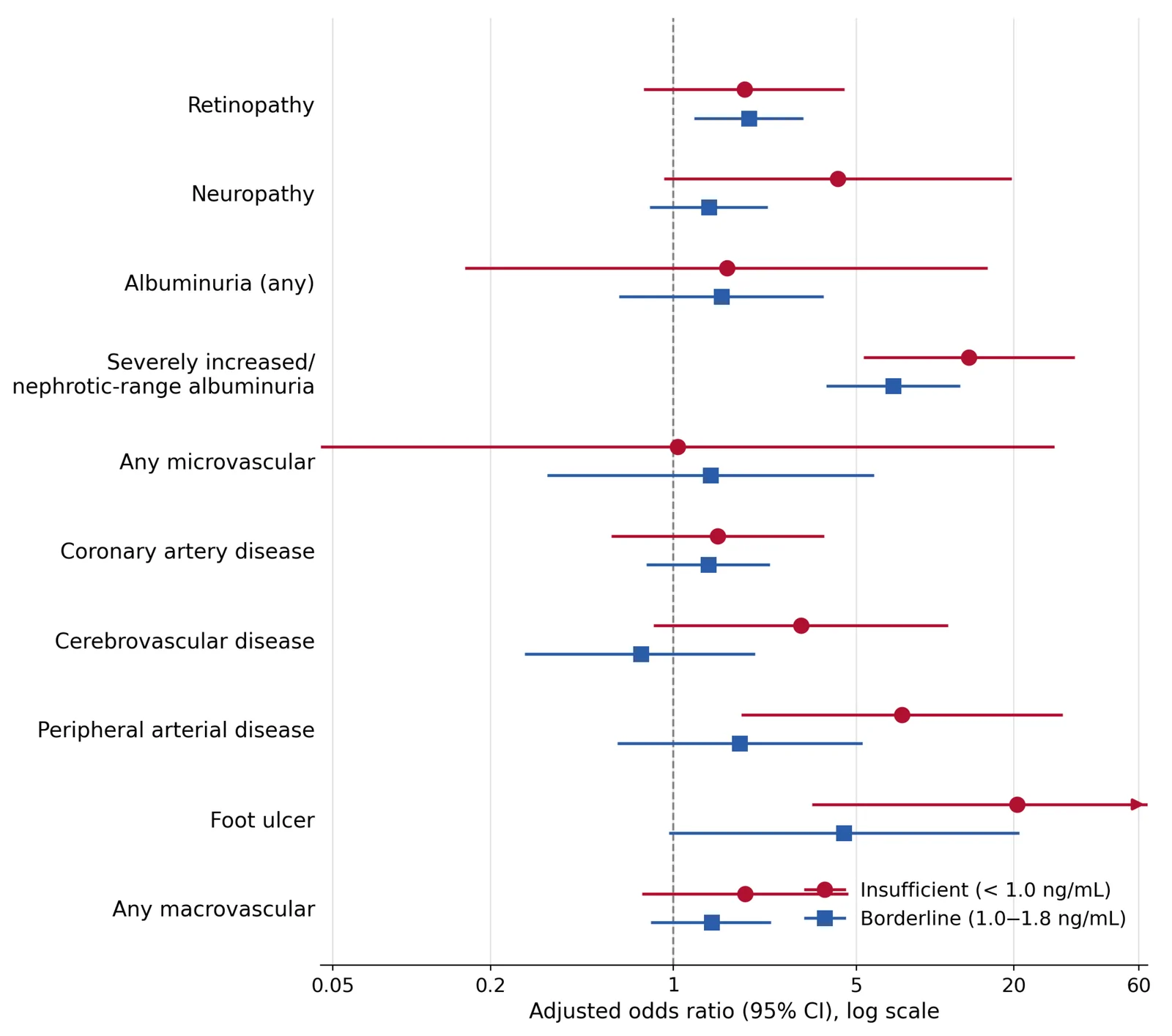
Adjusted odds ratios (95% CI) for microvascular and macrovascular complications by C-peptide groups, relative to the normal/high group. The odds ratios are adjusted for age, sex, duration of diabetes, HbA1c and BMI. Models for cerebrovascular disease, peripheral arterial disease, foot ulcer and the composite microvascular endpoint were fitted with Firth penalisation and adjusted for age and duration of diabetes only. The x-axis is logarithmic, and the dashed line marks an odds ratio of 1; an arrowhead indicates a confidence interval extending beyond the plotted range. Note the contrast between the two renal outcomes: the presence of albuminuria shows no independent association, whereas severely increased or nephrotic-range albuminuria shows the strongest association in the analysis.

Crude and adjusted associations between C-peptide groups and each complication are shown in Table 2 and summarised in Figure 2. In crude models, the insufficient and borderline groups had higher odds of essentially every complication than the normal/high group. After adjustment for age, sex, duration of diabetes, HbA1c and BMI, this uniformity disappeared, and a much more specific pattern emerged.

The two renal outcomes showed markedly different adjusted associations. C-peptide was not independently associated with the presence of albuminuria: relative to the normal/high group, the adjusted odds ratio was 1.60 (95% CI 0.16–15.86; *p* = 0.686) for the insufficient group and 1.53 (95% CI 0.62–3.74; *p* = 0.357) for the borderline group. By contrast, the association with albuminuria severity was strong and highly significant, with adjusted odds ratios for severely increased or nephrotic-range albuminuria of 13.47 (95% CI 5.33–34.07) in the insufficient group and 6.90 (95% CI 3.83–12.44) in the borderline group (both *p* < 0.001). In a sensitivity analysis additionally adjusting this model for CKD-EPI eGFR, the association was essentially unchanged (insufficient group: adjusted OR 12.50, 95% CI 4.80–32.55; and borderline group: adjusted OR 7.06, 95% CI 3.87–12.86), indicating that adjustment for renal function did not materially alter the association between C-peptide and albuminuria severity.

Among the remaining complications, borderline C-peptide remained independently associated with retinopathy (adjusted OR 1.94, 95% CI 1.20–3.13; *p* = 0.007), and insufficient C-peptide with peripheral arterial disease (adjusted OR 7.47, 95% CI 1.82–30.72; *p* = 0.005) and foot ulcer (adjusted OR 20.59, 95% CI 3.38–125.6; *p* = 0.001); these last two estimates are based on small numbers of events (24 and 14, respectively), are imprecise, and should be regarded as exploratory and hypothesis-generating rather than confirmatory. The associations with neuropathy, coronary artery disease, cerebrovascular disease and both composite endpoints were substantially attenuated and were no longer statistically significant after adjustment for diabetes duration and glycaemic control. The composite microvascular endpoint was uninformative because it was near-universal in this cohort (569 of 590 patients, 96.4%) and was fitted with Firth penalisation owing to quasi-complete separation.

Modelling C-peptide continuously gave a consistent picture (Table 3). Each 1-standard-deviation decrease in log-transformed C-peptide was independently associated with severely increased or nephrotic-range albuminuria (adjusted OR 2.20, 95% CI 1.62–3.00; *p* < 0.001) and with foot ulcer (adjusted OR 2.26, 95% CI 1.32–3.85; *p* = 0.003), and more weakly with retinopathy (OR 1.28, 95% CI 1.01–1.63) and neuropathy (OR 1.38, 95% CI 1.08–1.76), but not with the presence of albuminuria (OR 1.02, 95% CI 0.70–1.47) or with any macrovascular endpoint. The association with the total number of complications was of borderline significance (adjusted incidence rate ratio 1.07, 95% CI 1.00–1.14; *p* = 0.061).

### 3.6. ROC Analysis: Independence from Diabetes Duration, HbA1c and Glucose

To test directly whether C-peptide was associated with severe albuminuria independently of established risk factors, we compared a base logistic regression model containing diabetes duration, HbA1c and fasting plasma glucose with a model additionally including log-transformed C-peptide.

**Figure 3:**
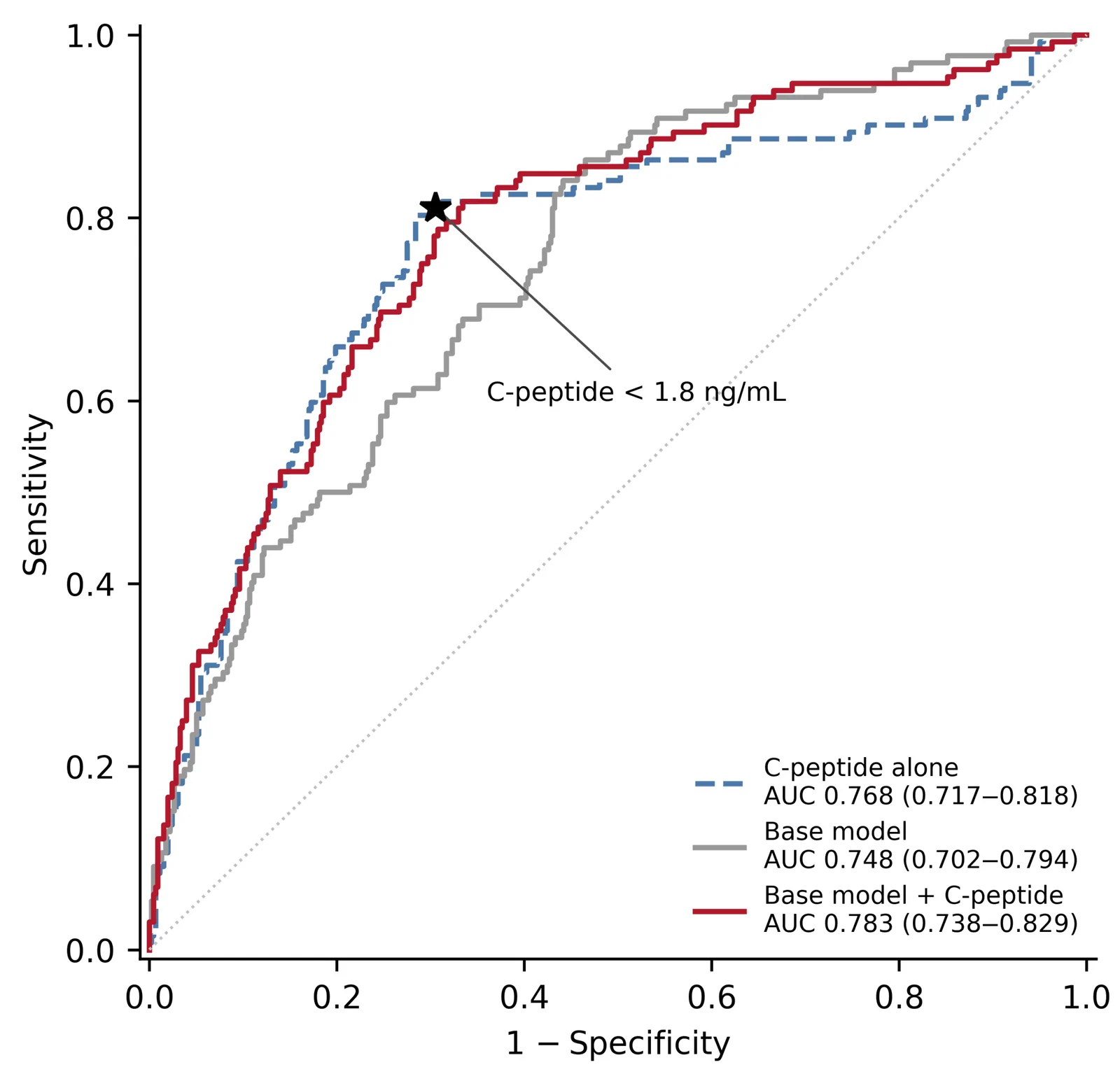
Receiver operating characteristic curves for severely increased or nephrotic-range albuminuria (versus normo- or microalbuminuria). The curves show discrimination by C-peptide alone, by the base model of diabetes duration, HbA1c and fasting plasma glucose, and by the base model plus log-transformed C-peptide. The star marks the Youden-optimal C-peptide cutoff, which was derived in this dataset and requires external validation. Adding C-peptide was associated with a statistically significant but modest improvement in discrimination (ΔAUC = 0.035, DeLong *p* = 0.027). AUC, area under the curve.

For severely increased or nephrotic-range albuminuria, C-peptide was independently associated with the outcome in the combined model (per 1-standard-deviation increase, adjusted OR 0.46, 95% CI 0.34–0.63; *p* < 0.001), independent of diabetes duration (adjusted OR 1.35, 95% CI 1.06–1.72; *p* = 0.017) and fasting plasma glucose (adjusted OR 1.90, 95% CI 1.33–2.71; *p* < 0.001); HbA1c was not independently significant in this model (adjusted OR 0.75, 95% CI 0.52–1.09; *p* = 0.130). By ROC analysis (Figure 3), the base model discriminated severe albuminuria, with an AUC of 0.748 (95% CI 0.702–0.794). Adding C-peptide increased the AUC to 0.783 (95% CI 0.738–0.829), a statistically significant but modest improvement by using DeLong’s test (ΔAUC = 0.035, *p* = 0.027); because statistical significance does not by itself establish clinical utility, and calibration or decision-curve analysis was not performed, this increment should not be interpreted as demonstrating clinically meaningful improvement in discrimination. Notably, C-peptide alone discriminated severe albuminuria at least as well as the three-variable base model (AUC 0.768, 95% CI 0.717–0.818). The optimal C-peptide cutoff, by the Youden index, was <1.80 ng/mL (sensitivity 81.1%, specificity 69.4%), closely matching the normal/high threshold used to define our C-peptide groups. Because this cutoff was derived and evaluated using the same dataset, this agreement should not be taken as evidence that 1.80 ng/mL is a validated, reproducible boundary; because internal cross-validation was not performed, its apparent performance may be optimistic, and external validation in an independent cohort is required before it can be considered for clinical use.

For the presence of albuminuria, by contrast, C-peptide added nothing: it was not significant in the combined model (adjusted OR 0.96, *p* = 0.806), and the AUC was essentially unchanged by its addition (0.745 to 0.745; ΔAUC < 0.001, *p* = 0.783). C-peptide alone discriminated this outcome poorly (AUC 0.659, 95% CI 0.595–0.724).

## 4. Discussion

In this cross-sectional study of 590 consecutively enrolled patients with T2DM, lower fasting serum C-peptide was associated in unadjusted analyses with a higher prevalence of both microvascular and macrovascular complications, as well as with worse glycaemic control, longer disease duration and poorer renal function. The central contribution of this study, however, lies in what survived adjustment. Once diabetes duration and glycaemic control were accounted for, the broad association with all complications collapsed to a specific one: low C-peptide marked the severity of diabetic kidney disease, not its presence, and did not consistently mark macrovascular disease at all.

This distinction matters because it has been obscured in much of the existing literature. Patients with low C-peptide have had diabetes for longer and control it less well, and both of those facts independently drive end-organ damage. A crude association between C-peptide and, say, coronary artery disease is, therefore, close to uninformative. In our data, the crude odds ratio for coronary artery disease in the insufficient group was 2.40, but the adjusted estimate was 1.47, with a confidence interval spanning unity; the same pattern held for cerebrovascular disease and for both composite endpoints. Reporting the crude figures alone would have supported a conclusion that our own adjusted analysis does not sustain.

Although C-peptide has long been regarded only as an indicator of endogenous insulin secretion, accumulating evidence shows that it is a biologically active peptide involved in microvascular regulation, endothelial function and inflammatory signalling. Chen et al. reported that C-peptide exerts protective effects on the microcirculation and endothelial integrity, suggesting that low levels may reflect both beta-cell dysfunction and the loss of these protective mechanisms [26]. Several mechanisms may explain the specifically renal association we observed. Brunskill and colleagues reported that C-peptide improves renal microcirculation, reduces glomerular hyperfiltration, and modulates inflammatory and fibrotic pathways [27], processes that are central to diabetic kidney disease and that plausibly operate at the stage of established, progressive glomerular injury rather than at the first appearance of microalbuminuria. Consistent with our findings, Jiang et al. found that patients with diabetic nephropathy had significantly lower serum C-peptide than those without, with an inverse relationship between C-peptide and the severity of renal impairment [28]. Because the kidney contributes substantially to the clearance of circulating C-peptide, impaired renal function would, if anything, be expected to raise rather than lower measured C-peptide; the inverse association we observed between low C-peptide and severe albuminuria is, therefore, unlikely to be explained by reduced renal clearance alone, although a causal interpretation cannot be established from cross-sectional data. Consistent with this, the association between C-peptide group and albuminuria severity was materially unchanged after additionally adjusting for CKD-EPI eGFR (Section 3.5), arguing against confounding by renal function as the principal explanation.

The dissociation between the presence and severity of albuminuria is, to our knowledge, an underappreciated feature of this relationship. Microalbuminuria was extremely common in our cohort, affecting more than two-thirds of patients, and its presence was largely determined by diabetes duration rather than by beta-cell reserve. Progression to severely increased or nephrotic-range albuminuria, by contrast, was strongly and independently graded by C-peptide, with a nearly sevenfold adjusted increase in odds even in the borderline group. Because our design is cross-sectional, these data show that lower C-peptide is associated with more advanced albuminuria at a single time point; they cannot establish that individual patients go on to progress to more advanced disease, and any prognostic language should be read in this associative rather than longitudinal sense. This suggests that C-peptide is not a screening marker for incipient nephropathy but may instead help discriminate which patients with established albuminuria have more advanced disease. The convergence of the data-derived Youden cutoff (1.80 ng/mL) with the a priori ADA-based threshold used to define our groups is consistent with, but does not by itself establish, a reproducible boundary; because the cutoff was derived and evaluated in the same dataset, its performance may be optimistic, and external validation in an independent cohort is required before it can be regarded as reproducible.

The relationship between C-peptide and diabetic complications has been examined in several previous studies with mixed results. Huang et al. reported that reduced serum C-peptide was significantly associated with an increased prevalence of both microvascular and macrovascular complications in T2DM [29], and a similar pattern was described by Kumar et al. [30]; both analyses, however, were largely unadjusted and are, therefore, closer to our crude than to our adjusted results. Sari and Balci, studying 318 patients with T2DM, found an association between serum C-peptide and macrovascular but not microvascular complications [31]—the opposite emphasis to ours. Marx and Walcher suggested that, under certain metabolic conditions, C-peptide may itself promote vascular inflammation and atherogenesis [32], highlighting that its vascular effects may be context-dependent and offering one explanation for why a consistent macrovascular signal has been difficult to demonstrate. A retrospective cohort of 1410 patients reported that higher C-peptide was associated with a reduced risk of microvascular complications [33], in line with the direction, though not the specificity, of our findings. This heterogeneity may partly reflect the dual biology of C-peptide with respect to vascular risk: in some studies on T2DM, higher C-peptide—itself a marker of insulin resistance and compensatory hyperinsulinaemia—has been linked to greater cardiovascular risk, whereas, in others, as in our unadjusted analyses, low C-peptide tracking with advanced beta-cell failure and long-standing disease appears to drive the crude association instead. Because most of the macrovascular associations in our own data were attenuated to non-significance after adjustment, our findings should not be read as evidence that C-peptide is vasoprotective; rather, they indicate that the crude macrovascular signal in this cohort was largely confounded by diabetes duration and glycaemic control, and the relationship between C-peptide and macrovascular disease should be considered unresolved.

Two further observations deserve comment. First, total cholesterol, LDL cholesterol and triglycerides were lower in the insufficient C-peptide group; this probably reflects the greater degree of insulin resistance, and consequently higher circulating insulin, in patients with normal or high C-peptide, rather than a favourable lipid phenotype in beta-cell failure. Second, in unadjusted ordinal regression, both ACEi/ARB and SGLT-2 inhibitor use appeared to be associated with more severe albuminuria. This is almost certainly confounding by indication—these agents are preferentially prescribed to patients who already have albuminuria—and both associations disappeared after adjustment. We report it because the crude estimate, taken at face value, would be seriously misleading, and because it illustrates the same interpretive problem that affects the crude C-peptide–complication associations.

Finally, insulin significantly moderated the inverse association between C-peptide and HbA1c, with the C-peptide–HbA1c relationship strongest at low insulin concentrations. This is consistent with the interpretation that C-peptide indexes glycaemic control most informatively when circulating insulin is low, that is, when beta-cell secretory reserve rather than insulin resistance is the limiting factor. The effect was small in absolute terms, and the model explained only 6% of the variance in HbA1c, so it should be regarded as mechanistically suggestive rather than clinically actionable.

### 4.1. Strengths and Limitations

This study has several limitations. The cross-sectional design precludes inferences about causality or temporal sequence; in particular, we cannot distinguish whether low C-peptide contributes to progressive albuminuria or whether both reflect a common, longer-standing disease process. The three C-peptide groups also differed significantly in age, which is itself a determinant of diabetic complications; although age was included as a covariate in all adjusted models, residual confounding by age cannot be fully excluded, and future studies could consider propensity-score matching to further reduce this imbalance. The single-centre setting may limit generalisability, and the cohort was unusual in the high prevalence of albuminuria, which leaves only 61 patients with normoalbuminuric and, therefore, limited precision for the “presence of albuminuria” comparison—the wide confidence intervals around those estimates should be read as uninformative rather than as evidence of no effect. C-peptide was measured at a single fasting time point, which may not fully capture dynamic or stimulated beta-cell function. The albuminuria category was likewise assigned from a single spot urine ACR measurement obtained at the study visit rather than from repeated measurements; because urinary albumin excretion can vary from day to day, this may have led to some misclassification, particularly around the microalbuminuria/severely increased-albuminuria boundary, which may have introduced non-differential outcome misclassification and potentially attenuated the observed associations. All complications other than albuminuria were ascertained from existing medical records rather than by protocol-driven examination at the study visit. This is likely to underestimate their true prevalence, since undiagnosed disease would be missed; it prevents any grading of severity; and it could introduce differential ascertainment if patients with more advanced disease had been investigated more frequently. The limitation applies with particular force to peripheral arterial disease, for which ankle–brachial index measurement was not performed, so that only clinically recognised disease could be captured. The renal outcomes, which rest on measurements obtained at the study visit, are not subject to this limitation, and it is on these that our principal conclusion rests. Renal function was assessed by albuminuria category and eGFR without renal biopsy, so non-diabetic kidney disease cannot be excluded. The number of events for peripheral arterial disease, foot ulcer and cerebrovascular disease was small, and although Firth penalisation mitigates small-sample bias, the corresponding estimates remain imprecise and should be considered hypothesis-generating. No formal sample-size calculation was performed, as this study enrolled all eligible consecutive patients during a fixed period. Finally, multiple complications were tested without formal correction for multiplicity; the renal severity finding is robust to any reasonable correction, but the borderline associations should be interpreted with that in mind.

The strengths of this study include a relatively large, real-world consecutive sample, a comprehensive assessment of metabolic, renal, and vascular parameters, the availability of a normoalbuminuric reference group allowing the presence and severity of kidney disease to be separated, and an analysis strategy that reports adjusted alongside crude estimates and tests the incremental discriminatory value formally, rather than relying on statistical significance alone.

### 4.2. Clinical Implications

C-peptide is an inexpensive and widely available measurement that, in this cohort, carried independent information about the severity of diabetic kidney disease and performed comparably to a three-variable clinical model in discriminating severe albuminuria, although the incremental improvement when C-peptide was added to that model was modest (ΔAUC = 0.035) and its clinical significance is unproven. It did not, however, identify which patients had albuminuria at all, and it should not be used for that purpose. Among patients with T2DM and established albuminuria, a fasting C-peptide below approximately 1.8 ng/mL may identify patients who could merit further evaluation or closer renal surveillance in future prospective studies; because this cutoff was derived and evaluated in the same dataset, its performance should be regarded as potentially optimistic pending external validation. These implications are hypothesis-generating and require prospective, externally validated confirmation before they inform practice.

### 4.3. Future Research Directions

Prospective, multicentre studies with longitudinal follow-up are needed to determine whether low C-peptide precedes and predicts progression to advanced diabetic kidney disease, and whether the 1.80 ng/mL cutoff identified here retains its discriminative performance in an independent cohort. Such studies should incorporate calibration and decision-curve analysis to establish whether C-peptide adds clinically meaningful value beyond statistical significance and should test its incremental value against a broader panel of established renal biomarkers. Mechanistic studies directly comparing renal C-peptide clearance with circulating concentrations across stages of diabetic kidney disease would help clarify whether the association observed here reflects a biologically protective role for C-peptide or is chiefly a marker of concurrent beta-cell failure.

## 5. Conclusions

In patients with T2DM, lower fasting serum C-peptide was associated, using unadjusted analyses, with a higher prevalence of microvascular and macrovascular complications. After adjustment for diabetes duration and glycaemic control, the independent association was confined largely to the severity of diabetic kidney disease: C-peptide was strongly associated with severely increased or nephrotic-range albuminuria but not with the presence of albuminuria, and macrovascular associations were not consistently independent. C-peptide may, therefore, provide information associated with advanced renal involvement in T2DM. Prospective, externally validated, multicentre studies are needed to clarify whether this relationship is causal and to define the potential protective role of C-peptide.

## Figures and Tables

**Table 1:** Demographic, clinical, laboratory and complication characteristics of patients with type 2 diabetes mellitus stratified by fasting serum C-peptide level.

Variable	Insufficient (<1.0 ng/mL) *n* = 35	Borderline (1.0–1.8 ng/mL) *n* = 212	Normal/High (≥1.8 ng/mL) *n* = 343	Overall (*n* = 590)	*p*-Value	Effect Size
Age, years	67.0 (61.0–70.5)	64.0 (57.8–70.0)	61.0 (54.0–69.0)	62.0 (55.0–70.0)	0.001 *^	
Sex, female	20 (57.1)	133 (62.7)	215 (62.7)	368 (62.4)	0.805	V = 0.03
BMI, kg/m^2^	29.3 (26.4–34.9)	29.0 (26.3–31.4)	29.2 (26.6–33.2)	29.1 (26.4–32.6)	0.280	
Duration of DM, years	17.0 (14.5–20.0)	13.0 (10.0–17.0)	7.0 (4.0–8.0)	8.0 (5.0–12.0)	<0.001 *^	
HbA1c, %	9.3 (7.8–11.1)	8.8 (7.1–10.8)	7.1 (6.5–8.4)	7.6 (6.6–9.6)	<0.001 *^	
FPG, mg/dL	199.0 (129.0–319.5)	181.5 (133.5–255.2)	143.0 (116.0–189.5)	157.0 (122.0–214.8)	<0.001 *^	
Urea, mg/dL	19.0 (13.8–21.6)	15.8 (12.4–20.4)	14.0 (11.1–17.1)	14.7 (11.5–19.0)	<0.001 *^	
Creatinine, mg/dL	0.90 (0.70–1.10)	0.80 (0.65–0.92)	0.75 (0.60–0.90)	0.80 (0.60–0.90)	0.035	
CKD-EPI eGFR, mL/min/1.73 m^2^	82.0 (56.1–96.5)	89.0 (69.0–99.6)	93.2 (78.1–102.1)	91.0 (73.5–101.3)	0.001 *^	
Total cholesterol, mg/dL	168.0 (136.0–217.0)	192.5 (161.0–224.5)	193.0 (168.5–224.0)	192.0 (165.0–224.0)	0.048 *	
LDL cholesterol, mg/dL	95.0 (72.0–130.5)	110.5 (89.0–136.0)	118.0 (98.0–142.0)	114.5 (95.0–139.0)	0.011 *	
HDL cholesterol, mg/dL	48.0 (45.5–57.0)	49.5 (41.0–59.2)	48.0 (40.5–57.0)	49.0 (41.0–58.0)	0.698	
Triglycerides, mg/dL	138.0 (90.5–192.0)	176.5 (123.0–255.0)	172.0 (127.0–240.5)	172.0 (123.0–242.0)	0.025 *#	
Magnesium, mg/dL	1.80 (1.60–1.90)	1.90 (1.70–2.00)	1.90 (1.80–2.00)	1.90 (1.70–2.00)	0.279	
Insulin, µIU/mL	3.3 (2.4–8.9)	6.2 (4.4–9.0)	14.6 (10.1–21.8)	10.4 (5.9–17.7)	<0.001 *^	
C-peptide, ng/mL	0.80 (0.50–0.80)	1.50 (1.20–1.60)	3.80 (2.95–5.70)	2.45 (1.50–4.10)	<0.001 *#^	
Albuminuria category, *n* (%)					<0.001	V = 0.35
Normoalbuminuria	1 (2.9)	10 (4.7)	50 (14.6)	61 (10.3)		
Microalbuminuria	13 (37.1)	116 (54.7)	268 (78.1)	397 (67.3)		
Severely increased albuminuria	10 (28.6)	69 (32.5)	23 (6.7)	102 (17.3)		
Nephrotic-range albuminuria	11 (31.4)	17 (8.0)	2 (0.6)	30 (5.1)		
Retinopathy	22 (62.9)	113 (53.3)	69 (20.1)	204 (34.6)	<0.001 *^	V = 0.36
Neuropathy	33 (94.3)	171 (80.7)	226 (65.9)	430 (72.9)	<0.001 *^	V = 0.20
Coronary artery disease	12 (34.3)	59 (27.8)	61 (17.8)	132 (22.4)	0.005 ^	V = 0.13
Cerebrovascular disease	7 (20.0)	10 (4.7)	13 (3.8)	30 (5.1)	0.002 #*	V = 0.17
Peripheral arterial disease	6 (17.1)	10 (4.7)	8 (2.3)	24 (4.1)	0.001 #*	V = 0.18
Foot ulcer	5 (14.3)	7 (3.3)	2 (0.6)	14 (2.4)	<0.001 #*	V = 0.21
Hypertension	24 (68.6)	127 (59.9)	193 (56.3)	344 (58.3)	0.313	V = 0.06
Antidiabetic regimen, *n* (%)					<0.001	V = 0.32
Single OAD	2 (5.7)	43 (20.3)	170 (49.6)	215 (36.4)		
Multiple OADs	11 (31.4)	112 (52.8)	144 (42.0)	267 (45.3)		
OADs + basal insulin	12 (34.3)	32 (15.1)	20 (5.8)	64 (10.8)		
OADs + basal–bolus insulin	5 (14.3)	15 (7.1)	6 (1.7)	26 (4.4)		
Basal–bolus insulin	5 (14.3)	7 (3.3)	3 (0.9)	15 (2.5)		
Basal insulin only	0 (0.0)	3 (1.4)	0 (0.0)	3 (0.5)		
Statin	12 (34.3)	82 (38.7)	112 (32.7)	206 (34.9)	0.350	V = 0.06
ACEi/ARB	15 (42.9)	94 (44.3)	146 (42.6)	255 (43.2)	0.919	V = 0.02
SGLT-2 inhibitor	16 (45.7)	111 (52.4)	98 (28.6)	225 (38.1)	<0.001 ^	V = 0.23

Continuous variables are presented as medians (25th–75th percentile) and were compared using the Kruskal–Wallis test with the Dunn–Bonferroni post-hoc test; categorical variables are presented as *n* (%) and were compared using the Pearson chi-square test, with Fisher’s exact test and Bonferroni correction for pairwise comparisons. #, * and ^ denote statistically significant pairwise differences (Bonferroni-adjusted *p* < 0.05) between the insufficient and borderline, insufficient and normal/high, and borderline and normal/high groups, respectively. V, Cramér’s V. BMI, body mass index, DM, diabetes mellitus, HbA1c, glycated haemoglobin, FPG, fasting plasma glucose, CKD-EPI, Chronic Kidney Disease Epidemiology Collaboration, eGFR, estimated glomerular filtration rate, LDL, low-density lipoprotein, HDL, high-density lipoprotein, OAD, oral antidiabetic drug, ACEi, angiotensin-converting enzyme inhibitor, ARB, angiotensin receptor blocker, SGLT-2, sodium–glucose co-transporter-2.

**Table 2:** Crude and adjusted associations between fasting serum C-peptide group and microvascular and macrovascular complications (reference: normal/high C-peptide, ≥1.8 ng/mL).

Complication/C-Peptide Group	Crude OR (95% CI)	*p*	Adjusted OR (95% CI)	*p*
Microvascular complications				
Retinopathy (*n* events = 204)				
Insufficient (<1.0)	6.72 (3.22–14.01)	<0.001	1.87 (0.77–4.50)	0.164
Borderline (1.0–1.8)	4.53 (3.11–6.61)	<0.001	1.94 (1.20–3.13)	0.007
Neuropathy (*n* events = 430)				
Insufficient (<1.0)	8.54 (2.01–36.22)	0.004	4.25 (0.92–19.62)	0.064
Borderline (1.0–1.8)	2.16 (1.44–3.25)	<0.001	1.37 (0.81–2.29)	0.239
Albuminuria (any category vs. normoalbuminuria) (*n* events = 529)				
Insufficient (<1.0)	5.80 (0.78–43.35)	0.087	1.60 (0.16–15.86)	0.686
Borderline (1.0–1.8)	3.45 (1.71–6.96)	<0.001	1.53 (0.62–3.74)	0.357
Severely increased/nephrotic-range albuminuria (vs. normo-/microalbuminuria) (*n* events = 132)				
Insufficient (<1.0)	19.08 (8.66–42.01)	<0.001	13.47 (5.33–34.07)	<0.001
Borderline (1.0–1.8)	8.68 (5.31–14.18)	<0.001	6.90 (3.83–12.44)	<0.001
Any microvascular complication ^a^ (*n* events = 569)				
Insufficient (<1.0)	4.27 (0.24–75.07)	0.321	1.04 (0.04–28.62)	0.980
Borderline (1.0–1.8)	5.06 (1.34–19.15)	0.017	1.39 (0.33–5.85)	0.652
Macrovascular complications				
Coronary artery disease (*n* events = 132)				
Insufficient (<1.0)	2.41 (1.14–5.11)	0.022	1.48 (0.58–3.77)	0.414
Borderline (1.0–1.8)	1.78 (1.18–2.68)	0.006	1.36 (0.79–2.33)	0.272
Cerebrovascular disease ^a^ (*n* events = 30)				
Insufficient (<1.0)	6.44 (2.42–17.18)	<0.001	3.07 (0.84–11.20)	0.089
Borderline (1.0–1.8)	1.27 (0.56–2.90)	0.572	0.75 (0.27–2.05)	0.575
Peripheral arterial disease ^a^ (*n* events = 24)				
Insufficient (<1.0)	8.70 (2.90–26.09)	<0.001	7.47 (1.82–30.72)	0.005
Borderline (1.0–1.8)	2.05 (0.81–5.15)	0.128	1.79 (0.61–5.27)	0.290
Foot ulcer ^a^ (*n* events = 14)				
Insufficient (<1.0)	24.63 (5.23–116.0)	<0.001	20.59 (3.38–125.6)	0.001
Borderline (1.0–1.8)	4.99 (1.18–21.13)	0.029	4.48 (0.96–21.02)	0.057
Any macrovascular complication (*n* events = 154)				
Insufficient (<1.0)	2.82 (1.38–5.79)	0.005	1.88 (0.76–4.65)	0.171
Borderline (1.0–1.8)	1.74 (1.18–2.57)	0.005	1.40 (0.82–2.36)	0.214

Odds ratios from binary logistic regression. Adjusted models include age, sex, duration of diabetes, HbA1c and BMI (*n* = 590). ^a^ Firth’s penalised-likelihood logistic regression with limited adjustment (age and duration of diabetes) was used because of a low event count or quasi-complete separation. OR, odds ratio, CI, confidence interval.

**Table 3:** Adjusted associations between continuously modelled fasting serum C-peptide and each complication.

Outcome	Adjusted OR or IRR per 1-SD Decrease in log C-Peptide (95% CI)	*p*
Retinopathy	1.28 (1.01–1.63)	0.044
Neuropathy	1.38 (1.08–1.76)	0.011
Albuminuria (any)	1.02 (0.70–1.47)	0.931
Severely increased/nephrotic-range albuminuria	2.20 (1.62–3.00)	<0.001
Any microvascular complication	1.29 (0.75–2.22)	0.357
Coronary artery disease	1.01 (0.78–1.31)	0.923
Cerebrovascular disease	0.96 (0.60–1.53)	0.867
Peripheral arterial disease	1.36 (0.83–2.22)	0.218
Foot ulcer	2.26 (1.32–3.85)	0.003
Any macrovascular complication	1.02 (0.80–1.31)	0.871
Total complication count (IRR)	1.07 (1.00–1.14)	0.061

C-peptide was natural-log transformed because of right skew and is expressed per 1-standard-deviation decrease (1 SD = 0.744 log ng/mL), so that an odds ratio above 1 indicates higher odds of the complication at lower C-peptide. The models are adjusted for age, sex, duration of diabetes, HbA1c and BMI, except for cerebrovascular disease, peripheral arterial disease, foot ulcer and the composite microvascular endpoint, which were fitted with Firth penalisation and adjusted for age and duration of diabetes only. The total complication count was modelled by Poisson regression and is reported as an incidence rate ratio. IRR, incidence rate ratio, OR, odds ratio, CI, confidence interval, SD, standard deviation.

## Data Availability

The data presented in this study are available on reasonable request from the corresponding author. The data are not publicly available because they contain information that could compromise the privacy of the research participants.

## Supplementary Materials

The following supporting information can be downloaded at: https://www.mdpi.com/article/10.3390/jcm15186950/s1. Table S1: Spearman rank correlations between fasting serum C-peptide and demographic, clinical, laboratory and complication variables. Table S2: Ordinal logistic regression for albuminuria severity. Table S3: STROBE statement—checklist of items that should be included in reports of cross-sectional studies. Reference [34] is cited in the Supplementary Materials.

## Abbreviations

The following abbreviations are used in this manuscript:

ACEi	Angiotensin-converting enzyme inhibitor
ACR	Albumin-to-creatinine ratio
ARB	Angiotensin receptor blocker
AUC	Area under the curve
BMI	Body mass index
CI	Confidence interval
CKD-EPI	Chronic Kidney Disease Epidemiology Collaboration
ECLIA	Electrochemiluminescence immunoassay
eGFR	Estimated glomerular filtration rate
FPG	Fasting plasma glucose
HbA1c	Glycated haemoglobin
HDL	High-density lipoprotein
IRR	Incidence rate ratio
LADA	Latent autoimmune diabetes in adults
LDL	Low-density lipoprotein
MODY	Maturity-onset diabetes of the young
OAD	Oral antidiabetic drug
OR	Odds ratio
ROC	Receiver operating characteristic
SGLT-2	Sodium–glucose co-transporter-2
T2DM	Type 2 diabetes mellitus
