# Supplementary material for: Low Serum C-Peptide and Albuminuria Severity in Type 2 Diabetes Mellitus: A Cross-Sectional Study
Source: J Clin Med. 2026 Sep 8;15(18):6950. doi: 10.3390/jcm15186950 (PMC13607856; doi:10.3390/jcm15186950)
Supplement: Supplementary file 1 [file jcm-15-06950-s001.zip › jcm-4511201-supplementary.pdf]

Supplementary Materials

Low Serum C-Peptide and Albuminuria Severity in Type 2 Diabetes Mellitus: A Cross-Sectional Study

Bektaş Işık and Bekir Tamer Tetiker

Table S1. Spearman rank correlations between fasting serum C-peptide and demographic, clinical, laboratory and complication variables.

Variable	Spearman ρ	p	n
Age, years	-0.169	< 0.001	590
BMI, kg/m^2	+0.061	0.137	590
Duration of DM, years	-0.636	< 0.001	590
HbA1c, %	-0.315	< 0.001	590
FPG, mg/dL	-0.210	< 0.001	590
Urea, mg/dL	-0.154	< 0.001	590
Creatinine, mg/dL	-0.052	0.210	590
CKD-EPI eGFR, mL/min/1.73 m^2	+0.129	0.002	590
Total cholesterol, mg/dL	-0.018	0.667	590
LDL cholesterol, mg/dL	+0.036	0.386	590
HDL cholesterol, mg/dL	-0.108	0.009	590
Triglycerides, mg/dL	+0.053	0.196	590
Magnesium, mg/dL	+0.053	0.200	590
Insulin, μIU/mL	+0.723	< 0.001	590
Albuminuria category	-0.397	< 0.001	590
Retinopathy	-0.338	< 0.001	590
Neuropathy	-0.211	< 0.001	590
Coronary artery disease	-0.097	0.019	590
Cerebrovascular disease	-0.074	0.073	590
Peripheral arterial disease	-0.100	0.015	590
Foot ulcer	-0.144	< 0.001	590
Total complication count	-0.359	< 0.001	590

Spearman's rank correlation coefficient (ρ); n = 590. A negative coefficient indicates that the variable increases as C-peptide falls. Albuminuria category was coded as an ordinal variable (0 normoalbuminuria, 1 microalbuminuria, 2 severely increased albuminuria, 3 nephrotic-range albuminuria). Total complication count is the sum of retinopathy, neuropathy, nephropathy, coronary artery disease, cerebrovascular disease, peripheral arterial disease and foot ulcer (range 0–7).

Table S2. Ordinal logistic regression for albuminuria severity.

Variable	Unadjusted OR (95% CI)	p	Adjusted OR (95% CI)	p
ACEi/ARB	1.74 (1.23–2.46)	0.002	1.07 (0.74–1.55)	0.719
SGLT-2 inhibitor	1.70 (1.20–2.41)	0.003	1.38 (0.94–2.03)	0.099
Age	–	–	1.01 (0.99–1.03)	0.377
Duration of DM	–	–	1.10 (1.07–1.14)	< 0.001
HbA1c	–	–	1.16 (1.07–1.25)	< 0.001
eGFR	–	–	0.97 (0.96–0.98)	< 0.001

Proportional-odds ordinal logistic regression with albuminuria as a four-level ordinal outcome (normoalbuminuria < microalbuminuria < severely increased albuminuria < nephrotic-range albuminuria); n = 590. An odds ratio above 1 indicates higher odds of a more severe albuminuria category. The unadjusted model included ACEi/ARB and SGLT-2 inhibitor use only; the adjusted model additionally included age, duration of diabetes, HbA1c and CKD-EPI eGFR. The apparent unadjusted association of both drug classes with more severe albuminuria is consistent with confounding by indication and did not persist after adjustment. Inspection of cut-point-specific coefficients indicated that the proportional-odds assumption was reasonably satisfied for the treatment variables but less well satisfied for eGFR, whose association was steepest at the transition from normo- to microalbuminuria.

Table S3. STROBE Statement—checklist of items that should be included in reports of cross-sectional studies.

Section	Item No	Recommendation	Reported on
Title and abstract	1a	Indicate the study's design with a commonly used term in the title or the abstract.	Title; Abstract (Methods)
	1b	Provide in the abstract an informative and balanced summary of what was done and what was found.	Abstract
Introduction — Background/rationale	2	Explain the scientific background and rationale for the investigation being reported.	Section 1, paragraphs 1–4
Introduction — Objectives	3	State specific objectives, including any prespecified hypotheses.	Section 1, final paragraph
Methods — Study design	4	Present key elements of study design early in the paper.	Section 2.1
Methods — Setting	5	Describe the setting, locations, and relevant dates, including periods of recruitment, exposure, follow-up, and data collection.	Section 2.1
Methods — Participants	6a	Give the eligibility criteria, and the sources and methods of selection of participants.	Section 2.2
Methods — Variables	7	Clearly define all outcomes, exposures, predictors, potential confounders, and effect modifiers. Give diagnostic criteria, if applicable.	Sections 2.3–2.6
Methods — Data sources/measurement	8*	For each variable of interest, give sources of data and details of methods of assessment (measurement). Describe comparability of assessment methods if more than one group.	Sections 2.3, 2.4, 2.5
Methods — Bias	9	Describe any efforts to address potential sources of bias.	Sections 2.4, 2.7, 4.1
Methods — Study size	10	Explain how the study size was arrived at.	Section 2.7; Section 4.1
Methods — Quantitative variables	11	Explain how quantitative variables were handled in the analyses. If applicable, describe which groupings were chosen and why.	Sections 2.6, 2.7
Methods — Statistical methods	12a	Describe all statistical methods, including those used to control for confounding.	Section 2.7
	12b	Describe any methods used to examine subgroups and interactions.	Section 2.7 (moderation analysis)
	12c	Explain how missing data were addressed.	Section 2.7 (data-checking paragraph); complete data for all 590 participants after correction

	12d	If applicable, describe analytical methods taking account of sampling strategy.	Not applicable (consecutive sampling of all eligible patients)
	12e	Describe any sensitivity analyses.	Sections 2.7 and 3.5 (eGFR-adjusted sensitivity analysis)
Results — Participants	13a	Report numbers of individuals at each stage of the study.	Sections 2.2 and 3.1 (n = 590 consecutive eligible patients)
	13b	Give reasons for non-participation at each stage.	Section 2.2 (exclusion criteria)
	13c	Consider use of a flow diagram.	Not included; single-visit consecutive enrolment with no loss to follow-up
Results — Descriptive data	14a	Give characteristics of study participants and information on exposures and potential confounders.	Table 1
	14b	Indicate the number of participants with missing data for each variable of interest.	Section 2.7 (data-checking paragraph)
Results — Outcome data	15*	Report numbers of outcome events or summary measures.	Tables 1–3
Results — Main results	16a	Give unadjusted estimates and, if applicable, confounder-adjusted estimates and their precision.	Tables 2 and 3
	16b	Report category boundaries when continuous variables were categorised.	Section 2.6
	16c	If relevant, translate estimates of relative risk into absolute risk for a meaningful time period.	Not applicable (cross-sectional design)
Results — Other analyses	17	Report other analyses done — e.g., subgroup, interaction, and sensitivity analyses.	Sections 3.4, 3.5, 3.6; Supplementary Tables S1–S2
Discussion — Key results	18	Summarise key results with reference to study objectives.	Section 4, opening paragraph
Discussion — Limitations	19	Discuss limitations of the study, taking into account sources of potential bias or imprecision. Discuss both direction and magnitude of any potential bias.	Section 4.1
Discussion — Interpretation	20	Give a cautious overall interpretation considering objectives, limitations, multiplicity of analyses, results from similar studies, and other relevant evidence.	Sections 4, 4.1, 4.2
Discussion — Generalisability	21	Discuss the generalisability (external validity) of the study results.	Section 4.1
Other information —	22	Give the source of funding and the role of the	Funding statement

Funding		funders for the present study.	

**Give information separately for exposure and outcome variables, as applicable. This checklist is based on the STROBE Statement for cross-sectional studies (von Elm E, Altman DG, Egger M, Pocock SJ, Gøtzsche PC, Vandenbroucke JP; STROBE Initiative. The Strengthening the Reporting of Observational Studies in Epidemiology (STROBE) statement: guidelines for reporting observational studies. Lancet 2007, 370, 1453–1457).*
